# The impact of viability assessment using cardiac MRI and echocardiogram on the outcome of revascularization in patients with multi-vessels coronary artery disease and moderate to severe ischemic cardiomyopathy

**DOI:** 10.15537/smj.2023.44.4.20220133

**Published:** 2023-04

**Authors:** Atif Alzahrani, Hani Mufti, Anas Alswat, Bsaim Altirkistani, Mohammed Aljehani, Ahmed Jazzar, Fahad Alutaibi, Amir Abushouk, Jamilah Al Rahimi, Wail Al Kashkari, Mohammed Althobaiti

**Affiliations:** *From King Faisal Cardiac Center (Alzahrani, Mufti, Al Rahimi, Al Kashkari), King Abdulaziz Medical City, Ministry of National Guard Health Affairs, Jeddah; from the Department of Radiology (Althobaiti), King Abdulaziz Medical City, Ministry of National Guard Health Affairs, Jeddah, from King Abdullah International Medical Research Center, (Alzahrani, Mufti, Alswat, Altirkistani, Aljehani, Jazzar, Alutaibi, Abushouk, Rahimi, Kashkari, Althobaiti), Jeddah, Kingdom of Saudi Arabia; from the College of Medicine (Alzahrani, Mufti, Alsawat, Altirkistani, Aljehani, Jazzar, Alutaibi, Abushouk, Al Rahimi, Al Kashkari, Althobaiti), King Saud Bin Aldulaziz University for Health Sciences, Jeddah, Kingdom of Saudi Arabia.*

**Keywords:** revascularization, viability assessment, cardiac mri, echo, ischemic cardiomyopathy

## Abstract

**Objectives:**

To investigate the influence of viability assessment in the management of patients with ischemic cardiomyopathy (ICM).

**Methods:**

This retrospective cohort study included all patients with ICM with moderate to severely reduced left ventricular ejection fraction (LVEF) who underwent viability assessment using cardiac magnetic resonance imaging (MRI) and echocardiogram as modalities of imaging. In addition, LVEF, modality of choice, and treatment plans were all extracted as main variables from the electronic database. One hundred 6 patients who met the inclusion criteria from December 2014 to December 2019 were included.

**Results:**

Posttreatment LVEF improved by 5% in the viable group compared to the nonviable group (*p*=0.016). Regardless of the treatment received, 6 (8.8%) patients in the viable group died due to cardiac causes after an 18-month follow-up period; in contrast, 7 (18.4%) patients died due to cardiac causes in the nonviable group. However, despite that difference, this was not statistically significant (*p*=0.153). Medical therapy alone was observed in 32 (84.2%) patients in the nonviable group compared to 32 (47.1%) in the viable group (*p*<0.001). Although the reduction in hospitalization for cardiac reasons was not statistically significant, the viable arm had 50% fewer hospitalizations than the nonviable arm (*p*=0.051).

**Conclusion:**

Patients with viable myocardium had better outcomes in which LVEF significantly improved posttreatment. Additionally, there was a reduction in the number of hospitalizations for cardiac reasons in the viable group compared to the nonviable group, even though the difference was not statistically significant. However, further studies with a larger number of patients are needed to determine a definite conclusion.

**I**schemic cardiomyopathy (ICM) is when the left ventricular systolic function is impaired due to significant coronary artery disease. Ischemic cardiomyopathy results from the reduction of blood supply to the myocardium that is caused by coronary artery diseases (CAD). The reduction of the myocardial blood flow can be acute secondary to myocardial infarction with ventricular remodeling or chronic by progressive narrowing of coronary arteries, which will reduce the ventricular contractile reserve and lead to a condition called hibernating myocardium.^1-3^

Ischemic cardiomyopathy is considered the most common cause of death worldwide. Moreover, CAD are the major contributor of deaths in ischemic cardiomyopathy, killing more than 370,000 people worldwide annually. In the United States, ICM accounts for 1 in every 4 deaths, killing one person every 36 seconds.^4^ Thus, 103 per 100,000 patients is the rate of death resulted from CAD.^1,4^ Regionally, 42% of deaths in Saudi Arabia are related to cardiac causes.^5^ The Prevalence of CAD in Saudi Arabia is estimated to be 5.5% in the whole population, with more Prevalence in males than females.^6^

When dealing with CAD, treatment aims to re-establish the blood supply and restore the ventricular function of the heart, either by revascularization using coronary artery bypass grafting (CABG), percutaneous coronary intervention (PCI) or by conventional medical therapy alone when the revascularization is not possible. When discussing revascularization, the term myocardial viability is defined as the myocardium in which the contractility of the heart is predicted to be improved or in which remolding or necrosis can be averted when the blood flow returns.^7^ Viability assessment of the myocardium is used to determine the extent of damage caused by the ischemic event; by predicting any improvement in the left ventricle ejection fraction (LVEF). Positron emission tomography (PET) is considered the gold standard for detecting myocardial viability; however, other modalities like cardiac MRI (CMR) and dobutamine echocardiography can also be used for the same purpose. The popularity of CMR and dobutamine echocardiography is mainly due to their availability and low cost compared to PET.^7,8^ According to the literature, some researchers stated that myocardial viability in patients with ischemic cardiomyopathy who underwent CABG has no predictive benefits, especially in the long term.^9^ However, other researches found that the viability assessment is valuable and beneficial as it decreases the cardiac event (myocardial infarction, cardiac death) and decreases the re-hospitalization for cardiac causes within one year.^10-12^ Since this topic is controversial, there is a need for more studies on myocardial viability assessment and whether it is beneficial for patients with ischemic cardiomyopathy in short and long-term aspects.

Our study measured the impact of viability assessment on outcomes in patients with multi-vessel coronary artery disease and ischemic cardiomyopathy. The primary objectives of this study were to determine the effect of viability assessment using cardiac MRI or echocardiography on reducing mortality, reducing hospitalization, and increasing the left ventricular ejection fraction (LVEF) within 18 months as a follow-up period.

## Methods

A retrospective chart review study with a cohort design was conducted at King Faisal Cardiac Center (KFCC), King Abdulaziz Medical City, Jeddah, Saudi Arabia. The inclusion criteria includes adult patients with CAD who had done viability assessment from December 2014 to December 2019. In addition, patients with mildly reduced LVEF (LVEF >/=45%) and patients under the age of 18 years were all excluded from the study as per exclusion criteria. Using the electronic hospital medical records system, there were 106 patients from which the data was collected, using a consecutive sampling technique.

The data was obtained from the hospital’s electronic records using the Best Care 2.0 System. A data collection sheet that contained the study variables was used. These variables included demographics data such as age, gender, height, weight, and nationality. Other variables included pre and post-assessments of LVEF as percentages, state of living, number of hospitalizations for cardiac-related causes, results of viability assessment, LV size, modality of imaging. They were it either CMR or echocardiography, other cardiac risk factors and medications usage, and lastly, the plan of treatment and whether it was PCI, CABG, or medical therapy alone. As the study was conducted retrospectively, along with the sampling technique used, which was a consecutive one, there was no need for informed consent; therefore, Institutional Review Board approval was used instead. Then, these data were collected and entered manually on the excel sheet by the research team. The validity and quality of the collected data were ensured by the principal investigator of the research.

### Statistical analysis

Characteristics of patients who underwent viability, based on the above-mentioned inclusion criteria, were evaluated. Because of the small sample size, non-parametric measures of central tendency and variance were used. Continuous variables were reported using the median and interquartile range and were compared using the Wilcoxon rank-sum test. Categorical variables were reported as frequencies and percentages and were analyzed by Chi-square or Fisher’s exact test as appropriate (Fisher’s exact test when more than 20% of cells have expected frequencies <5. The Kruskal-Wallis test was used for ordinal attributes. All statistical tests were 2-tailed, and *p*-values of <0.05 were considered significant. Statistical analyses were performed using R software, version 4.0.2. (R Foundation for Statistical Computing, Vienna, Austria).

## Results

A total of 106 subjects who met the inclusion criteria were included in the study. Out of these, viable myocardium and non-viable myocardium were respectively reported to be 68 (64.2%) vs. 38 (35.8%). The median age of the viable group was 66 years (interquartile range 58-74 years), while 68 years (inter-quartile range 60-73 years) was the median for the non-viable group. Myocardial infarction, as a history of cardiac pathology, was presented in 21 (55.3%) of the non-viable group, which was significantly higher than the viable group 21 (30.9%) (*p*=0.013). The detailed demographic characteristics are reported in Table 1.

**Table 1:** - Patients demographic characteristics (N=106).

Characteristic	All patients (n=106)	Viable (n=68)	Non-viable (n=38)	*P*-value
Age (years), median (IQR)	66 (59-73)	66 (58-74)	68 (60-73)	0.586
Gender - Male	95 (89.6)	61 (89.7)	34 (89.5)	0.896
BMI (kg/m^2^), median (IQR)	28.3 (25-32)	28.6 (24.8-32.1)	28.1 (25.3-30.8)	0.661
Nationality - Saudi	105 (99.1)	68 (100)	37 (97.4)	0.179
***Comorbidities***				
DM	91 (85.8)	57 (83.8)	34 (89.5)	0.423
HTN	82 (77.4)	53 (77.9)	29 (76.3)	0.848
DLP	66 (62.3)	38 (55.9)	28 (73.7)	0.069
Smoking	29 (27.4)	20 (29.4)	9 (23.7)	0.526
***Cardiac pathology***				
MI	42 (39.6)	21 (30.9)	21 (55.3)	0.013*
CABG	10 (9.4)	6 (8.8)	4 (10.5)	0.743
PCI	14 (13.2)	7 (10.3)	7 (18.4)	0.238
***Medications***				
ARB or ACEI	89 (84.0)	58 (85.3)	31 (81.6)	0.617
BB	93 (87.7)	61 (89.7)	32 (84.2)	0.408
Nitrates	45 (42.5)	30 (44.1)	15 (39.5)	0.643
Loop diuretics	80 (75.5)	51 (75.0)	29 (76.3)	0.877
MRA	60 (56.6)	41 (60.3)	19 (50.0)	0.305

Values as presented as number and percentages (%). IQR: interquartile range, BMI: body mass index, DM: diabetes mellitus, HTN: hypertension, DLP: dyslipidemia, MI: myocardial Infarction, CABG: coronary artery bypass graft, PCI: Percutaneous coronary interventionm ARB: angiotensin receptor blockers, ACEI: angiotensin converting enzyme inhibitors, BB: beta blockers, MRA: mineralocorticoid receptor antagonists. *Significant *p*-value.

In the viable group, when it comes to therapeutic modalities, medical therapy alone was considered in 32 (47.1%) of patients, CABG was presented in 22 (32.4%), and only 14 (20.6%) underwent PCI. In the non-viable group, on the contrary, medical therapy alone was reported in 32 (84.2%) of cases (*p*<0.001). Collectively, CABG, and PCI represented only 6 (15.8%) of the patients. The survival rate after 18 months of follow-up was higher in viable myocardium compared to the non-viable (56 [82.4%] vs. 25 [65.8%]) even though this difference was not statistically significant (*p*=0.153). Cardiac-related outcomes are summarized in Table 2.

**Table 2:** - Imaging modalities, treatment and cardiac outcomes based on the myocardial viability status.

Characteristic	All patients (n=106)	Viable (n=68)	Non-viable (n=38)	*P*-value
***Modality***				
ECHO	10 (9.4)	6 (8.8)	4 (10.5)	
MRI	95 (89.6)	61 (89.7)	34 (89.5)	0.728
Nuclear	1 (0.9)	1 (1.5)	0 (0)	
***Treatment***				
CABG	24 (22.6)	22 (32.4)	2 (5.3)	
Medical	64 (60.4)	32 (47.1)	32 (84.2)	<0.001*
PCI	18 (17)	14 (20.6)	4 (10.5)	
Admissions for cardiac cause, median (IQR)	0.5 (0-1)	0 (0-1)	1 (0-2)	0. 051
Dilated heart	65 (61.3)	42 (61.8)	23 (60.5)	0.898
***Status***				0.153
Alive	81 (76.4)	56 (82.4)	25 (65.8)	
Died due to cardiac cause	13 (12.3)	6 (8.8)	7 (18.4)	
Died due to non-cardiac cause	12 (11.3)	6 (8.8)	6 (15.8)	
***LVEF***				
LVEF at baseline, median (IQR)	30 (25-36)	30 (25-37)	31 (25-35)	0. 817
LVEF post treatment, median (IQR)	33 (27-40)	35 (30-42)	30 (25-35)	0. 016*
LVEDI, median (IQR)	75 (59-89)	76 (59-89)	71 (62-95)	0. 957
Creatinine clearance, median (IQR)	68 (56-82)	70 (54-81)	67 (59-82)	0. 935

Values are presented as number s and percentages (%). IQR: interquartile range, ECHO: echocardiography, MRI: magnetic resonance imaging, CABG: coronary artery bypass graft, PCI: Percutaneous coronary intervention, LVEF: left ventricular ejection fraction, LVED: left ventricular end diastolic index, *Significant *p*-value.

When medical therapy alone was considered as a therapeutic modality, 7 (21.9%) of the non-viable group died due to cardiac causes compared to the viable group 2 (6.2%). Furthermore, the viable group had a median of 0.5 visits due to cardiac reasons; on the other hand, a median of 1 visit was in the non-viable group. Thus, the viable group reported fewer visits by at least 50% during the follow-up period even though this reduction was not statistically significant (*p*=0.532). Characteristics of the medical therapy group are presented in Table 3.

**Table 3:** - Characteristics and outcome of patients who received medical therapy alone based on viability status.

Characteristic	All patients (n=106)	Viable (n=68)	Non-viable (n=38)	*P*-value
Gender - Male	57 (89.1)	29 (90.6)	28 (87.5)	0.971
Admissions for cardiac cause, median (IQR)	1 (0-1)	0.5 (0-1)	1 (0-1.3)	0.532
***Status***				0.141
Alive	46 (71.9)	26 (81.2)	20 (62.5)	
Died due to cardiac cause	9 (14.1)	2 (6.2)	7 (21.9)	
Died due to non-cardiac cause	9 (14.1)	4 (12.5)	5 (15.6)	
***LVEF***				
LVEF at Baseline, median (IQR)	30 (25-35)	30 (25.4-34.2)	31 (24.8-35)	0. 930
LVEF Post treatment, median (IQR)	32 (25-37.3)	34 (30.3-38.1)	30 (25-35.8)	0. 094

Values are presented as number s and percentages (%). LVEF= Left ventricular ejection fraction.

**Figure 1:**
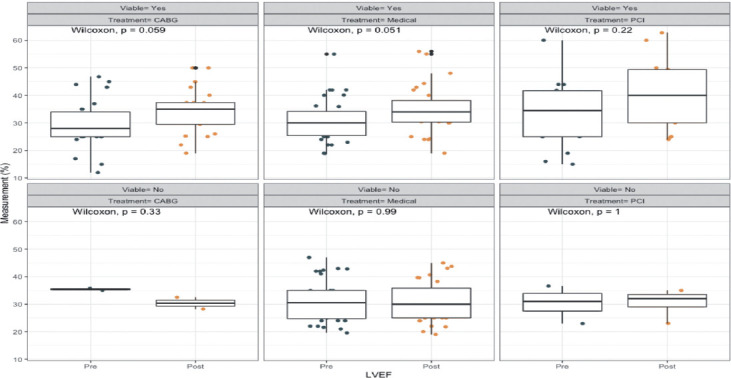
- Left ventricular ejection fraction by treatment modality and viability.

For those who had viable myocardium, overall LVEF was improved and significantly increased by 5% (*p*=0.016) post-treatment regardless of which therapy was performed. However, in patients in the non-viable group, therapeutic modalities did not play a positive role in terms of LVEF improvement. More demonstrations regarding LVEF with different treatment modalities are presented in (Figure 1).

## Discussion

When planning coronary revascularization, the primary purpose is to restore the blood supply to the ischemic myocardium.^13^ Multiple imaging modalities have been used to detect viable myocardium and predict functional recovery, including echocardiography, cardiac MRI, and PET.^7,14^ Given that there are controversies surrounding the current utility of viability assessment, this study aimed to evaluate the impact of viability assessment on the outcomes of revascularization in patients with CAD-ICM.

Our results showed that patients with viable myocardium underwent more revascularization strategies with CABG or PCI when compared to the non-viable group and showed relatively fewer cardiac-related deaths, events, and hospitalization, even though this difference was not statistically significant. Moreover, LVEF improved by 5% in patients with viable myocardium in comparison with the non-viable. One of the possible explanations is that viable but dysfunctional myocardium has the ability to recover when blood flow is restored. Also, revascularization has been shown to improve survival in viable myocardium.^15,16^ On the other hand, non-viable myocardium is associated with poorer outcomes, and any further reduction in blood supply or increased oxygen demand in these non-viable regions will eventually lead to re-infarction or fatal arrhythmia, increasing the mortality in non-viable patients, regardless of which treatment plan was chosen.^16,17^ In patients with non-viable myocardium, more previous myocardial infarctions were reported. These prior infarcts could leave the myocardium in an irreversibly damaged state and the formation of myocardial scars in the non-viable group.^16^

In comparison to another study, similar results were implicated, suggesting that the presence of myocardial infarction is associated more with non-viable myocardium.^18^ After assessing the myocardial viability by the 3 used imaging modalities, it was suggestive that going for medical treatment alone did not show any significant effect on the mortality rate of both the viable and non-viable groups. Yet, this finding contradicted prior studies, which state that medical therapy alone has a significant effect on increasing the mortality rate in patients with viable myocardium by 158% rather than both.^19^ It appears that there is no clear beneficial outcome when comparing between the pre and post-treatment effect of using medical treatment, CABG, or PCI as a route of treatment on the LVEF for the non-viable myocardium; nevertheless, a significant increase in the LVEF on the viable group was present regardless what therapy the patients were on. Also, choosing revascularization as a treatment route resulted in a better predicting outcome of the post-treatment effect on the LVEF with the patients who were in the viable group (Figure 1), which would support the results of prior studies stating that there is a better overall outcome of revascularization on the patients with viable myocardium.^10,17,19^ Adding to a previous study, which states that there is a relative increase of hospitalization for cardiac causes in terms of going for medical therapy as a route of treatment and in this study, it expands it to elucidate that patient with viable myocardium and using medical therapy showed a decreased incidence of hospitalization contrary to the non-viable group, but it is still not statistically significant.^20^

### Study limitations

Determining the need for viability assessment has been a challenge for cardiac specialists when discussing patients with ischemic cardiomyopathy. There were some limitations in this study. First, this study is a single-center study, and it only involved patients at KFCC in Jeddah. The second limitation, this study is a retrospective electronic chart review. A third limitation is that the number of patients in this study is small, making it difficult to find statistically significant differences between the 2 groups of patients. Given our sample size and with the assumption of a moderate effect size of myocardial viability on mortality (calculated effect size=0.57388), we calculated our study power of 80.8% at a significance level of 0.05 that is adequate for such study design.^21^ Additional research with a prospective design involving multi centers with a larger sample size is needed to shed light on viability assessment utility.

In conclusion, the current study suggests that patients with viable myocardium could have relatively less mortality and hospitalization. However, significant LVEF improvement can be observed if they adhere to the viability assessment recommendations. Conversely, patients with non-viable myocardium generally tend to have worse outcomes regardless of the treatment given, implicating the need to balance each treatment modality’s risks and benefits in those patients. Further research with a larger sample size is needed to investigate the viability assessment and its role in managing ischemic cardiomyopathy patients.
